# Exploring the Determinants of Treatment Completion Among Youth Who Received Medication-Assisted Treatment in the United States

**DOI:** 10.3390/healthcare13070798

**Published:** 2025-04-02

**Authors:** Esther A. Adeniran, Megan Quinn, Ying Liu, Billy Brooks, Robert P. Pack

**Affiliations:** 1 Cedars-Sinai Cancer, Cedars-Sinai Medical Center, Los Angeles, CA 90048, USA; 2 Department of Biostatistics and Epidemiology, College of Public Health, East Tennessee State University, Johnson City, TN 37614, USA; quinnm@etsu.edu (M.Q.); liuy09@etsu.edu (Y.L.); brooksb1@etsu.edu (B.B.); 3 Department of Community and Behavioral Health, College of Public Health, East Tennessee State University, Johnson City, TN 37614, USA; packr@etsu.edu

**Keywords:** adolescent, youth, Medication-Assisted Treatment of Opioid Use Disorder, opioid-related disorders, treatment episode data set (TEDS), treatment completion, treatment dropout

## Abstract

Background: An effective treatment for Opioid Use Disorder is Medication-Assisted Treatment (MAT). However, in the United States (US), this is still being underutilized by youth. Research indicates the need to develop strategies to reduce treatment barriers among these youth. Thus, we explored the rates of treatment completion and dropout among youth receiving MAT in US substance use treatment facilities and examined factors associated with treatment completion and dropout. Methods: This study used the 2019 Treatment Episode Data Set—Discharges. Our analysis was restricted to youth (12–24 years) who primarily used heroin at admission. Log-binomial regression was used to examine various determinants of treatment completion and dropout, guided by Andersen’s Behavioral Model. Results: The final sample size was 4917. Among youth reporting heroin use with receipt of MAT, those showing a higher likelihood of treatment completion than dropout were males [ARR: 1.23; 95% CI: 1.088–1.381; *p* = 0.0008], had 1–7 times [ARR: 1.33; 95% CI: 1.115–1.584; *p* = 0.0015] and 8–30 times self-help group participation [ARR: 1.50; 95% CI: 1.246–1.803; *p* < 0.0001], had co-occurring mental and substance use disorders [ARR: 1.28; 95% CI: 1.133–1.448, *p* < 0.0001], were admitted to detoxification [ARR: 2.80; 95% CI: 2.408–3.255; *p* < 0.0001] and residential/rehabilitation settings [ARR: 2.05; 95% CI: 1.749–2.400; *p* < 0.0001], and were located in the Midwest/West [ARR: 1.18; 95% CI: 1.030–1.358; *p* = 0.0173]. Conversely, other races (excluding Whites and Blacks/African Americans) [ARR: 0.75; 95% CI: 0.609–0.916; *p* = 0.0051], those who used MAT [ARR: 0.81; 95% CI: 0.694–0.946; *p* = 0.0077], and youth in the South [ARR: 0.45; 95% CI: 0.369–0.549; *p* < 0.0001] were less likely to report treatment completion than dropout. Conclusions: The majority of youth receiving MAT dropped out of treatment. We identified various factors that should be prioritized to address youth underutilization of MAT in the US.

## 1. Introduction

Opioid Use Disorder (OUD) is a pattern of maladaptive use of opioids (prescribed or illicit), which significantly impairs health or function [1]. Adolescents and young adults have a high prevalence of risky opioid use, OUD, and associated harms [2,3]. Given that OUD often begins during adolescence and young adulthood, access to effective treatment in this population is needed to prevent the progression of addiction and reduce long-term harm [4]. Scientific evidence identifies Medication-Assisted Treatment (MAT) as an effective treatment for OUD, but it is still being underutilized by youth [5,6,7,8,9]. MAT involves treating OUD using medications like methadone, buprenorphine, and/or naltrexone in conjunction with behavioral therapy/counseling [5,10,11]. Multiple studies have investigated the effectiveness of MAT for OUD among youth [4,12,13,14,15], confirming its role in improving abstinence from opioid use or recovery from OUD, improving retention, and reducing relapse. However, only about 28% of adolescents who need MAT receive it, and there is an average delay of four to seven years between the onset of OUD and the initiation of treatment [11].

Research shows that younger age is a predictor of treatment dropout among patients who are dependent on opioids [16,17,18]. Dropping out of MAT increases the risk of opioid use relapse [19,20], while treatment completion is associated with positive outcomes [21,22,23,24]. A prior study [18] emphasized the importance of identifying factors associated with treatment completion and dropout among youth who received MAT from substance use treatment (SUT) facilities in the United States (US). Furthermore, reports suggest that the coronavirus pandemic has increased substance use, opioid overdose, and relapse rates, thus setting back efforts to reduce the opioid epidemic [25,26,27,28]. A national survey found that among the 57.2 million individuals (≥12 years) who reported past-year illicit drug use in 2019, over 740,000 used heroin [29]. Despite possible reductions in heroin use over time [30], it is imperative that current users reach the end of the treatment program. Heroin has been a significant contributor to opioid-related overdose deaths among adolescents over time, although there is currently a rise in fatalities associated with illicit synthetic opioids like fentanyl [31,32]. Pre-pandemic data could provide insights into MAT utilization trends among youth who use heroin to improve access to life-saving treatment for this vulnerable population.

There is still a pressing need to develop strategies that decrease treatment barriers among youth with OUD [2,11,12]. To achieve this, further exploration of determinants of MAT completion and dropout is needed among this population. Andersen’s Behavioral Model (ABM) for Health Services is a conceptual framework useful for clarifying why people use health services, including SUT, and it comprehensively examines predisposing, enabling, need, and environmental factors [33,34,35,36]. The literature suggests factors associated with treatment completion and dropout in SUT facilities, including MAT, but most have targeted adults [18,37] instead of youth, and this gap remains largely unaddressed even after a decade. Demographic characteristics like age, sex, race, and education were found to be associated with treatment completion/dropout [19,36,38,39], and, in the present study, these demographics are considered predisposing factors (i.e., exist prior to the onset of an illness/are unlikely to change) based on the ABM [33,34,35]. Also, insurance, social support, previous SUT episodes, days waiting to enter treatment, and type of treatment service/setting [36,38,40,41,42] are suggested factors associated with treatment completion/dropout and are considered enabling factors (i.e., resources impacting health service use) [33,34,35]. Previous literature suggests that substance use problems and mental health treatment may be associated with treatment completion/dropout [18,36,41]; guided by the ABM, these were considered need factors (i.e., need for care as evaluated by a professional or perceived by the affected individual). The ABM suggests that environmental factors may consist of physical, political, and economic conditions, as well as the health care system [34,35,36,43], while the treatment environment is identified as associated with dropout or retention [44,45]. Based on the existing literature and guided by the ABM, the aforementioned potential factors or candidate variables were considered for examination in the current study.

Therefore, the current study utilized publicly available data to address two aims: (1) determine the rate of treatment completion and dropout among younger patients (12–24 years old) who received MAT in US SUT facilities and (2) identify the factors associated with treatment completion and dropout among our target population. Furthermore, we are not aware of any prior study that has utilized the ABM to examine multilevel determinants of treatment completion and dropout among our target population. This study could inform the development of policies and enable practitioners to develop and implement effective youth-based programs to improve treatment retention and completion rates, outcomes, and prognosis and save costs.

## 2. Materials and Methods

### 2.1. Data Source

Data used for this study were obtained from the 2019 Treatment Episode Data Set—Discharges (TEDS-D) (*n* = 1,722,503). TEDS-D is a publicly available data set annually collected by the Substance Abuse and Mental Health Services Administration (SAMHSA) and contains information on individuals ≥ 12 years in state-licensed or certified substance abuse treatment facilities that receive federal funding in the US. More details regarding TEDS-D can be found elsewhere [46]. The inclusion criteria for this study were those whose age at admission was <25 years old, used heroin as a primary substance at admission, and reported the reason for discharge as treatment completed and dropout. This study received an exemption from the East Tennessee State University Institutional Review Board.

### 2.2. Measures

#### 2.2.1. Dependent Variable: Treatment Completion and Dropout

The main outcome of interest was SUT completion and dropout, and this was derived from the variable “reason for discharge”. This had the following responses: (1) treatment completed, (2) dropped out of treatment, (3) terminated by facility, (4) transferred to another treatment program or facility, (5) incarcerated, (6) death, and (7) others (client transferred or discontinued treatment due to life circumstances). This was recoded as a dichotomous variable (treatment completed and dropout). Cases with other discharge reasons (i.e., 3–7) were excluded because of uncertainty about their treatment completion status and the focus of this study.

#### 2.2.2. Covariates

##### Predisposing Factors

These factors included age, sex, race, and educational level. Age (<25 years) was categorized as 12–17 and 18–24 years old. Sex was coded as male and female. Participants’ race included (1) Alaska Native (Aleut, Eskimo, Indian), (2) American Indian (other than Alaska Native), (3) Asian or Pacific Islander, (4) Black or African American, (5) White, (6) Asian, (7) other single race, (8) ≥2 races, and (9) Native Hawaiian/other Pacific Islander. We recoded race as (1) Black or African American, (2) White, and (3) other races. Educational level initially encompassed the following responses: (1) less than one school grade, no schooling, nursery school, kindergarten to grade 8, (2) grade 9 to 11, (3) grade 12 (or GED), (4) 1–3 years of college, university, or vocational school, and (5) 4 years of college/university, Bachelor of Arts/Bachelor of Science, some postgraduate study, or more. For this study, responses 1–3 were categorized as ≤high school, 4 as some college degree, and 5 as college graduate or more.

##### Enabling Factors

These included health insurance, self-help group participation, previous SUT episodes, days waiting to enter the treatment facility, and type of treatment service setting. Participants’ responses to having health insurance included (1) private insurance, Blue Cross/Blue Shield, and Health Maintenance Organization (HMO), (2) Medicaid, (3) Medicare/others (such as TRICARE (the US military health care program for service members, retirees, and their families) and CHAMPUS (the Civilian Health and Medical Program of the Uniformed Services), now referred to as TRICARE), and (4) none. We categorized health insurance as a dichotomous variable (yes or no). Pertaining to past-month self-help group participation, options included (1) no attendance, (2) 1–7 times, and (3) 8–30 times. Previous SUT episodes was dichotomous (yes or no). Days waiting to enter a treatment facility had initial responses of 0, 1–7 days, 8–14 days, 15–30 days, and ≥31 days. This was recoded as ≤30 days and >30 days. Types of treatment services received at admission were recoded as detoxification (24 h hospital inpatient/free-standing residential), residential/rehabilitation (hospital, short-term, and long-term), and ambulatory (outpatient and ambulatory detoxification).

##### Need Factors

These included the use of MAT and having co-occurring mental health diagnosis and substance use disorders (SUDs). Clients’ use of MAT was dichotomous (yes/no) and denotes that they received opioid medications, such as methadone, buprenorphine, and/or naltrexone. Co-occurring mental and SUDs was dichotomous (yes or no).

##### Environmental Factor

This was addressed using the variable “census region” and was coded as Northeast, Midwest/West, and South.

### 2.3. Statistical Analysis

Descriptive statistics included frequencies and percentages. Bivariate associations were assessed using chi-square tests. To assess the relationships between independent variables and the outcome, we conducted multivariable log-binomial regression. The independent variables included predisposing factors (age, sex, race, and educational level), enabling factors (self-help group participation, previous SUT episodes, and the type of treatment/service setting at admission), need-based factors (use of MAT and having co-occurring mental and SUDs), and an environmental factor (census region). We chose log-binomial regression over logistic regression because our study’s outcome was common, and we were interested in reporting the adjusted risk ratios (ARR) [47]. The variables were included in the multivariable analysis if they met the threshold of *p* ≤ 0.20 [48]. A backward selection process was then applied [49], retaining variables with *p* ≤ 0.05. Multicollinearity among the variables in the final model was evaluated using the variance inflation factor, and no issues were detected. SAS version 9.4 (SAS Institute, Cary, NC, USA) was used to conduct all statistical analyses.

## 3. Results

### 3.1. Population Characteristics and Bivariate Associations

Table 1 shows the descriptive statistics and bivariate associations of our study population (*n* = 4917) in which the overall proportion of those who completed treatment (51.5%) was greater than dropouts (48.5%). Notably, we found that regarding youth who received MAT, 43.9% completed treatment, while 56.1% dropped out. Of our total sample, 98.8% were 18–24 years old, 55.6% were males, and 85.5% were White. In particular, youth who completed treatment were mostly males (54.0%), White (52.3%), and had some college/vocational school education (55.3%). Among youth who completed treatment, 51.9% had health insurance, while 50.1% did not participate in self-help groups. Overall, 79% of our sample reported experiencing ≥ 1 previous SUT episodes. We found that 98% of youth who completed treatment and reported treatment dropout waited ≤ 30 days to enter the SUT facility. Additionally, 38.7% of youth reported the type of treatment or service setting at admission as ambulatory. Among those who completed treatment, 53.1% had co-occurring mental and SUDs, and 55.2% were located in the Northeast. In the bivariate analyses, health insurance and days waiting to enter substance abuse treatment facility were not significantly associated with the outcome.

### 3.2. Factors Associated with Treatment Completion and Dropout

Table 2 presents the multivariable log-binomial regression for factors associated with treatment completion compared to dropout among youth. Compared to females, males [ARR: 1.23; 95% CI: 1.088–1.381; *p* = 0.0008] showed a higher likelihood of treatment completion. Compared to Whites, other races [ARR: 0.75; 95% CI: 0.609–0.916; *p* = 0.0051] were 25% less likely to report treatment completion. Compared to those with no self-help group participation in the past month, those reporting one to seven times self-help group participation [ARR: 1.33; 95% CI: 1.115–1.585; *p* = 0.0015] were 1.33 times as likely to complete treatment. Likewise, youth reporting 8–30 times self-help group participation [ARR: 1.50; 95% CI: 1.246–1.803; *p* < 0.0001] were 1.5 times as likely to complete treatment. Youth who reported the type of treatment or setting at admission as detoxification [ARR: 2.80; 95% CI: 2.408–3.255; *p* < 0.0001] and residential/rehabilitation [ARR: 2.05; 95% CI: 1.749–2.400; *p* < 0.0001] had a higher likelihood of treatment completion than those in ambulatory settings. Compared to youth who did not receive MAT, those with receipt of MAT [ARR: 0.81; 95% CI: 0.694–0.946; *p* = 0.0077] were associated with a lower likelihood of treatment completion. Compared to those without co-occurring mental and SUDs, those with these diagnoses had increased likelihood of treatment completion [ARR: 1.28; 95%CI: 1.133–1.448; *p* < 0.0001]. Finally, compared to youth in the Northeast, those located in the Midwest and West were 1.18 times as likely to complete treatment [ARR: 1.18; 95% CI: 1.030–1.358; *p* = 0.0173]. However, youth located in the South [ARR: 0.45; 95% CI: 0.369–0.549; *p* < 0.0001] were 55% less likely to report treatment completion.

## 4. Discussion

Prior research indicates that treatment completion and retention are essential in addressing SUD, especially among youth who are opioid dependent and are more susceptible to treatment dropout [3,16,22]. We found that among youth who received MAT, treatment dropout was more prevalent than treatment completion (56.1% vs. 43.9%). Previous studies indicate the underutilization of MAT by youth in the US [5,8,11], and the present study shows that underutilization is occurring even in subsidized treatment facilities. Furthermore, we found that youth with receipt of MAT (categorized as a need factor) were associated with a lower likelihood of treatment completion than dropout. While prior research has shown that the use of medications for OUD increases the likelihood of treatment completion in short-term residential treatment programs, it reduces the likelihood of treatment completion in long-term residential treatment programs [50]. Given that our study includes multiple treatment settings or services at admission, this variability may have influenced our observed association pertaining to MAT use. Beyond the receipt of MAT, we identified several factors associated with our outcome, including sex, race, self-help group participation, type of treatment or service setting at admission, having co-occurring mental disorders and SUDs, and census region. Importantly, our study underscores the need to address barriers to treatment completion for youth already receiving MAT and to improve MAT use in US SUT facilities. The literature highlights barriers to the utilization of MAT [8,11,51,52], which are not fully applicable to treatment completion because the term “underutilization” is used interchangeably with lack of access. Some barriers include stigma, lack of support from friends and family, limited targeted care for youth in SUT facilities, practitioners having inadequate training or inexperience with medication administration, and commercial insurers not being subject to similar federal requirements as Medicaid that cap out-of-pocket costs [8,51,53,54].

As there is a need to develop strategies that limit treatment barriers among youth with OUD [2,11,12], this study adds to the literature by identifying significant factors associated with treatment completion and dropout while guided by ABM. Thus, this study could inform policy and programmatic efforts to improve treatment completion and retention rates among youth receiving MAT. The present study found that regarding predisposing factors, males were more likely to complete treatment compared to females. A prior study discovered no significant association between gender and attrition [18], while another study indicated that younger females have a lower likelihood of treatment retention than males [55]. Our findings imply that youth receiving MAT experience sex-based differences in treatment completion. An explanation could be that females receiving MAT tend to face numerous vulnerabilities, including a high prevalence of infectious diseases, sexual abuse, suicide, disparities in treatment needs, and higher perceived stigmatization when seeking help for substance use [56,57,58]. Due to the inconsistencies in the literature and limited studies focusing on youth, future studies should corroborate this sex-based finding. Conversely, compared to Whites, youth identifying as other races (constituting several racial minorities excluding Blacks/African Americans) reported a lower likelihood of treatment completion. This agrees with research that shows that younger individuals identifying as racial/ethnic minorities reported a lower likelihood of receipt of MAT [8,59]. Our study may be one of the first to determine racial differences in treatment completion and dropout among youth receiving MAT. This result may be explained by the presence of structural racism, racial discrimination in treatment facilities, and the need to develop and encourage the widespread adoption of culture-based treatment interventions [60,61,62]. Qualitative research is needed to better understand why racial minorities are mostly affected.

Pertaining to enabling factors, reporting 1–7 and 8–30 times of self-help group participation in the past month (referent = none) was positively associated with treatment completion. This finding is similar to that of an existing study that recorded high completion rates among adolescents with OUD receiving group therapy alongside MAT [63]. An adult-based study shedding light on combining self-help group participation with MAT use indicates that this could be beneficial to patients [64]. Additionally, a recent study found that MAT paired with self-help group participation increases the likelihood of treatment completion than MAT use alone [65]. It is imperative to promote self-help group participation among youth receiving MAT to enhance treatment completion.

A previous study [18] randomized youth into two treatment groups: group 1 received 12 weeks of buprenorphine/naloxone medication and psychosocial treatment, while group 2 received only 2 weeks of detoxification with buprenorphine/naloxone medication and 12 weeks of psychosocial treatment. Dropout rates differed notably between the groups. In group 1, dropout rates were 0% before week 2, 8% before week 4, and 28% before week 12, while group 2 had dropout rates of 8% before week 2, 36% between 2 and 4 weeks, and 81% before week 12. Thus, continuing buprenorphine/naloxone medication over a longer period (>2 weeks) can help improve treatment retention [18], even for those in detoxification. This finding is different from that in our study, which found that youth who received MAT and were admitted to 24 h detoxification settings were more likely to complete treatment than drop out. Likewise, the lowest dropout rate was found in 24 h detoxification (8.8%) rather than residential/rehabilitation (15.8%) and ambulatory (75.4%) settings. These findings may be due to the short detoxification period, detoxification occurring in a wide range of settings, and cost-effective insurance that covers a full range of detoxification services [66]. Still, detoxification is limited in that it may not encompass comprehensive substance abuse treatment [66]. Therefore, youth in detoxification settings require intensive treatment and longer treatment periods to recover from OUD [20].

Furthermore, youth receiving MAT in residential/rehabilitation settings (referent = ambulatory) were 2.05 times more likely to report treatment completion. This result is contrary to a study revealing a reduced likelihood of treatment completion among younger clients receiving treatment in residential programs [67]. Another study using a similar data set to ours found that those in long-term residential treatment and using OUD medications reported a lower likelihood of treatment completion, while retention was not significantly increased [50]. Notably, for those in short-term residential treatment, there was an increased likelihood of treatment completion and retention [50]. Similarly to our results, individuals (≥18 years) in residential treatment were more likely to report treatment completion, which could be due to the highly structured nature of this treatment setting [67]. Overall, we recognize that there is a need to keep youth in treatment, by preventing dropout and promoting successful completion. Additionally, we found that youth with co-occurring mental disorders and SUDs (referent = those without) were more likely to complete treatment. This aligns with the literature indicating that younger individuals who received buprenorphine for OUD without counseling demonstrated higher retention, improved opioid abstinence, and better psychiatric and psychosocial functioning [15]. Furthermore, evidence suggests that youth receiving MAT often also receive medications for mental health conditions [68,69]. This underscores the importance of integrated care, which simultaneously treats co-occurring disorders and has been shown to enhance treatment engagement and mental health outcomes [68].

Pertaining to the environment, this study found that youth in the Midwest and Western US were more likely to complete treatment than those in the Northeast. Also, this study demonstrates that youth in the South were less likely to complete treatment than drop out. However, limited research exists to corroborate these results. A closely related study reported that facilities in the South were 76% less likely to provide medication for OUD than those in the Northeast; also, those in the West had 85% less likelihood [70]. A systematic review further highlights the lack of access to medications for OUD in the South compared to other regions in the US [52]. We did not find a significant association between health insurance and treatment completion. Thus, we suggest that future studies explore this relationship, as expanding health insurance coverage may have a double impact of increasing treatment access and completion rates in Southern regions. More federal and state efforts are needed to improve not only treatment access but also completion rates among youth in the South [8].

The present study has additional policy implications. This study reveals that youth in subsidized treatment facilities hardly use MAT, and the few receiving treatment are vulnerable to dropping out. Despite federal initiatives, such as the 2016 21st Century Cures Act, which allocated about USD 1 billion to states for a period of 2 years to expand MAT provisions (e.g., treatment access) and build practitioners’ capacity [8,71], significant gaps remain [72,73]. Future funding efforts should adopt a long-term approach and could prioritize youth currently receiving MAT in subsidized SUT facilities to ensure treatment completion. Policies and funding agencies should prioritize vulnerable subgroups of youth, e.g., females and racial minorities, as they may be most affected by treatment dropout [60]. Since the pandemic, the Consolidated Appropriations Act has eliminated the federal requirement for practitioners to obtain a special waiver to prescribe buprenorphine, which reflects progress. However, recent research highlights the need to expand the number of practitioners qualified to prescribe medications [11], which would greatly benefit youth. Adequate training for practitioners and the development of youth-tailored interventions should be prioritized [11,37,60]. Future research using post-pandemic data can build upon the patterns uncovered by our study to further understand evolving trends and inform targeted interventions.

This study has some limitations. TEDS-D data do not comprise all admissions and discharges to US SUT facilities, especially non-publicly funded programs. Our sample comprises many treatment settings but is not specifically designed for MAT because it lacks data on Opioid Treatment Programs (OTPs) or Office-Based Opioid Treatment (OBOT). Likewise, this analysis was restricted to youth (<25 years) who received and did not receive MAT, primarily used heroin at admission, and reported the reason for discharge as treatment completed and dropout. This substantially reduced our sample size. Thus, our results are not generalizable to the entire population of youth receiving MAT in other US facilities. Also, TEDS-D data are not client-specific, indicating that these data may contain multiple individual records of discharges as well as admissions. Our findings should be interpreted with caution, i.e., on the basis of treatment discharges and not by person. Additionally, the interpretation of findings regarding other races should be approached with caution, as it includes both youth identifying as a single race and those with two or more races, limiting specificity to racial minorities. As stated earlier, we did not examine the impact of health insurance, as bivariate analyses showed a nonsignificant relationship with the outcome. Research indicates that Medicaid and the Children Health Insurance Program (CHIP) cover many youth and could improve youth access to MAT [51,74]. Moving forward, studies should examine the influence of health insurance on treatment completion and dropout among youth receiving MAT.

## 5. Conclusions

The majority of youth with a history of MAT dropped out of treatment. The receipt of MAT was associated with a lower likelihood of treatment completion in SUT facilities. Males showed a higher likelihood of completing treatment, while other races (excluding Whites and Blacks/African Americans) reported a lower likelihood. Enabling factors, including past month self-help group participation and being admitted to detoxification and residential/rehabilitation, were positively associated with treatment completion. Having co-occurring mental disorders and SUDs (a need factor) was associated with a higher likelihood of treatment completion. Pertaining to the environment, those in the Midwest/West were more likely to complete treatment, while those located in the South were less likely to complete treatment. These are important factors to consider when developing/implementing policy and programmatic interventions to improve treatment retention and completion rates among youth receiving MAT. Certain policy- or practice-based implications of our study entail adequately trained and available practitioners, addressing racial and sex-based disparities among youth in treatment, and improving the rates of youth receiving MAT in publicly funded SUT facilities in the US.

## Figures and Tables

**Table 1:** Characteristics of clients by treatment completion and dropout (*n* = 4917).

Variable	Treatment Completion and Dropout			
	Total *n* (%)	Completed Treatment *n* = 2533 (51.5%)	Dropped Out *n* = 2384 (48.5%)	*p*-Value
Predisposing Factors				
*Age*				
12–17 years	60 (1.2)	39 (65.0)	21 (35.0)	**0.0355**
18–24 years	4857 (98.8)	2494 (51.4)	2363 (48.6)	
*Sex*				
Male	2735 (55.6)	1477 (54.0)	1258 (46.0)	**<0.0001**
Female	2182 (44.4)	1056 (48.4)	1126 (51.6)	
*Race*				
Black or African American	275 (5.6)	112 (40.7)	163 (59.3)	**0.0010**
White	4204 (85.5)	2198 (52.3)	2006 (47.7)	
Other races	438 (8.9)	223 (50.9)	215 (49.1)	
*Educational level*				
≤High school	4367 (88.8)	2239 (51.3)	2128 (48.7)	**0.0165**
Some college/vocational school	497 (10.1)	275 (55.3)	222 (44.7)	
College graduate/postgraduate school/more	53 (1.1)	19 (35.9)	34 (64.1)	
Enabling Factors				
*Health insurance*				
Yes	3420 (69.5)	1776 (51.9)	1644 (48.1)	0.3792
No	1497 (30.5)	757 (50.6)	740 (49.4)	
*Past month self-help group participation*				
*No*	3633 (73.9)	1820 (50.1)	1813 (49.9)	**0.0035**
*1–7 times*	668 (13.6)	368 (55.1)	300 (44.9)	
*8–30 times*	616 (12.5)	345 (56.0)	271 (44.0)	
*Previous substance use treatment episodes*				
*Yes*	3865 (78.6)	2023 (52.3)	1842 (47.7)	**0.0263**
*No*	1052 (21.4)	510 (48.5)	542 (51.5)	
*Days waiting to enter substance abuse treatment facility*				
≤30 days	4819 (98.0)	2478 (51.4)	2341 (48.6)	0.3566
>30 days	98 (2.0)	55 (56.1)	43 (43.9)	
*Type of treatment or service setting at admission*				
24 h detoxification	1906 (38.8)	1184 (62.1)	722 (37.9)	**<0.0001**
Residential and rehabilitation	1107 (22.5)	613 (55.4)	494 (44.6)	
Ambulatory	1904 (38.7)	736 (38.7)	1168 (61.3)	
Need Factors				
*Use of MAT*				
Yes	1062 (21.6)	466 (43.9)	596 (56.1)	**<0.0001**
No	3855 (78.4)	2067 (53.6)	1788 (46.4)	
*Co-occurring mental and substance use disorders*				
Yes	2284 (46.5)	1214 (53.1)	1070 (46.9)	**0.0324**
No	2633 (53.5)	1319 (50.1)	1314 (49.9)	
Environment				
*Census region*				
Northeast	2343 (47.7)	1293 (55.2)	1050 (44.8)	**<0.0001**
Midwest and West	1901 (38.6)	1022 (53.8)	879 (46.2)	
South	673 (13.7)	218 (32.4)	455 (67.6)	

Note. MAT = Medication-Assisted Treatment; column percentage was reported only for total; variables with *p* ≤ 0.20 (bolded in the table) were eligible for inclusion in the full multivariable model; thus, health insurance and days waiting to enter substance abuse treatment facility were excluded.

**Table 2:** Multivariable log-binomial regression showing the relationship between independent variables and outcome (treatment completion vs. dropout).

Variable	Adjusted Model		
	ARR	95% CI	*p*-Value
Predisposing Factors			
*Age (12–17 years vs. 18–24 years)*	†		
*Sex (male vs. female)*	1.23	1.088–1.381	**0.0008**
*Race (ref = White)*			
Black or African American	0.84	0.646–1.096	0.2004
Other races	0.75	0.609–0.916	**0.0051**
*Educational level (ref = college graduate or more)*			
≤High school	†		
Some college degree	†		
Enabling Factors			
*Health insurance*	◊		
*Past month self-help group participation (ref = no)*			
1–7 times	1.33	1.115–1.585	**0.0015**
8–30 times	1.50	1.246–1.803	**<0.0001**
*Previous substance use treatment episodes* *(yes vs. no)*	†		
*Days waiting to enter substance abuse treatment facility*	◊		
*Type of treatment or service setting at admission (ref = ambulatory)*			
24 h detoxification	2.80	2.408–3.255	**<0.0001**
Residential and rehabilitation	2.05	1.749–2.400	**<0.0001**
Need Factors			
*Use of MAT (yes* vs. *no)*	0.81	0.694–0.946	**0.0077**
*Co-occurring mental and substance use disorders (yes vs. no)*	1.28	1.133–1.448	**<0.0001**
Environment Factor			
*Census region (ref = Northeast)*			
Midwest and West	1.18	1.030–1.358	**0.0173**
South	0.45	0.369–0.549	**<0.0001**

Note. Variables with *p* ≤ 0.05 are considered statistically significant and are bolded in the table; ARR: adjusted relative risk; CI: confidence interval; REF = reference; MAT = Medication-Assisted Treatment; †: originally included in the full model but dropped out of the final model; ◊: not included in the full model due to insignificant bivariate association.

## Data Availability

The data presented in this study are openly available in SAMHSA’s Treatment Episode Data Files website at https://www.datafiles.samhsa.gov/dataset/teds-d-2019-ds0001-teds-d-2019-ds0001, accessed 1 September 2021.

## Abbreviations

The following abbreviations are used in this manuscript:

MAT	Medication-Assisted Treatment
OUD	Opioid Use Disorder
SUD	Substance use disorder
SUT	Substance use treatment
TEDS-D	Treatment Episode Data Set—Discharges
US	United States
ARR	Adjusted risk ratios
CHIP	Children Health Insurance Program
